# Around the Hospitals

**Published:** 1893-03-04

**Authors:** 


### Around the Hospitals.

**Bradford Infirmary.**—It is satisfactory to notice that the Committee of the Bradford Infirmary, in their report just submitted to the annual meeting, draw attention to the criticism of our Commissioner, which we published on December 17th, 1892. The report admits "that the justice of the criticism, though candid, cannot be altogether denied," and adds that "some of the changes suggested are certainly desirable." Unfortunately, although Mr. Bacchus, who seconded the adoption of the report, declared "the mortuary to be absolutely a disgrace to the infirmary and the town," and that whenever any scheme of alteration was taken in hand it should not be a question of patching up the building, but of pulling down and entirely reconstructing it, he added that the necessary work could not, for the time being at any rate, be entertained. We are strongly of opinion that the insanitary "boxes," which now constitute the only conveniences attached to the larger wards in the older portions of the building, and also the mortuary, should be condemned and demolished without a moment's delay. Otherwise, although the Bradford Infirmary undoubtedly possesses many good points, it must be content to take a back place among the medical institutions of the country, especially when compared with those of Leeds and other neighbouring towns.

**St. Bartholomew's Hospital** is now able to set public apprehension at rest, and has officially stated that its sanitation has been most carefully revised and all defects removed. This has been accomplished at a cost of £6,397, and the work of the past six months since the completion of the alterations shows that the money has been well spent. It is a matter for congratulation that the reproach has been removed from an institution accomplishing such a large amount of excellent work as does St. Bartholomew's. No less than 5,953 inpatients, 16,143 out, and 142,745 casualty patients have been treated during the past year. The comfort of the patients is well looked after, and very marked improvements have taken place in the comfort of the nurses during the past few years. The nursing home, which was some four years back a disgrace to any institution, has been immensely improved, and an excellent library added, which is quite the pride of the nurses.

**The Edinburgh Hospital and Dispensary for Women and Children.**—Fountainbridge presents a most encouraging report for the past year. Dr. Sophia Jex-Blake, whose name will always be interesting as a pioneer amongst medical women, is attending medical officer to the institution. Free beds are felt to be a great and increasing want, and funds are urgently needed to secure one or two which would be available for some of the worst cases and the poorest of the dispensary patients. During the past year there have been 225 patients on the books of the dispensary, 154 of them being provident members, each of whom subscribes ten shillings a-year to defray the cost of medicines. The remaining 71 have been casual patients who attend at the dispensary for a period not exceeding three months. The work in connection with the cottage hospital, which was opened seven years ago, has also been satisfactory. Thirty-nine patients have been treated, and of these some have been cases which, as a rule, are seldom admitted into a general hospital. Much useful work could be done if larger funds were at the disposal of the committee.

